# Prevalence of *Chlamydia trachomatis* infection in women, heterosexual men and MSM visiting HIV counselling institutions in North Rhine-Westphalia, Germany - should Chlamydia testing be scaled up?

**DOI:** 10.1186/s12879-016-1915-2

**Published:** 2016-10-26

**Authors:** Anne Lallemand, Viviane Bremer, Klaus Jansen, Stine Nielsen, Dieter Münstermann, Andreas Lucht, Carsten Tiemann

**Affiliations:** 1 Robert Koch Institute, Berlin, Germany; 2 European Programme for Intervention Epidemiology Training (EPIET), ECDC, Stockholm, Sweden; 3 Labor Krone, Bad Salzuflen, Germany

**Keywords:** Chlamydia trachomatis, Prevalence, HIV test, STI

## Abstract

**Background:**

Patients asking for a free anonymous HIV test may have contracted other sexually transmitted infections (STIs) such as *Chlamydia trachomatis,* yet Chlamydia prevalence in that population is unknown. This study aimed to assess the prevalence and factors associated with Chlamydia infection in patients seeking HIV testing at local public health authorities (LPHA) in order to evaluate whether Chlamydia testing should be routinely offered to them.

**Methods:**

We conducted a cross-sectional study among patients (≥18 years) attending 18 LPHA in North Rhine-Westphalia from November 2012 to September 2013. LPHA collected information on participants’ socio-demographic characteristics, sexual and HIV testing behaviours, previous STI history and clinical symptoms. Self-collected vaginal swabs and urine (men) were analysed by Transcription-Mediated Amplification. We assessed overall and age-stratified Chlamydia prevalence and 95 % confidence intervals (95 % CI). Using univariate and multivariable binomial regression, we estimated adjusted prevalence ratios (aPR) to identify factors associated with Chlamydia infection.

**Results:**

The study population comprised 1144 (40.5 %) women, 1134 (40.1 %) heterosexual men and 549 (19.4 %) men who have sex with men (MSM); median age was 30 years. Chlamydia prevalence was 5.3 % (95 % CI: 4.1–6.8 %) among women, 3.2 % (95 % CI: 2.2–4.4) in heterosexual men and 3.5 % (95 % CI: 2.1–5.4) in MSM. Prevalence was highest among 18–24 year-old women (9 %; 95 % CI: 5.8–13) and heterosexual men (5.7 %; 95 % CI: 3.0–9.8 %), respectively. Among MSM, the prevalence was highest among 30–39 year-olds (4.4 %; 95 % CI: 1.9–8.5 %). Among those who tested positive, 76.7 % of women, 75.0 % of heterosexual men and 84.2 % of MSM were asymptomatic. Among women, factors associated with Chlamydia infection were young age (18–24 years versus ≥ 40 years, aPR: 3.0, 95 % CI: 1.2–7.8), having had more than 2 partners over the past 6 months (ref.: one partner, aPR: 2.1, 95 % CI: 1.1–4.0) and being born abroad (aPR: 1.9, 95 % CI: 1.0–3.5). Among heterosexual men, young age was associated with Chlamydia infection (18–24 years versus ≥ 40 years, aPR: 4.1, 95 % CI: 1.3–13). Among MSM, none of the variables were associated with Chlamydia infection.

**Conclusions:**

LPHA offering HIV tests should consider offering routine Chlamydia testing to women under 30 years. Women with multiple partners and those born abroad may also be considered for routine testing. Our results also suggest offering routine Chlamydia testing to heterosexual men under 25 years old. For MSM, we cannot draw specific recommendations based on our study as we estimated the prevalence of urethral Chlamydia infection, leaving out rectal and pharyngeal infections.

## Background

*Chlamydia trachomatis* (hereafter referred to as Chlamydia) is the most common bacterial STI in Europe [1]. In 2012, the rate of diagnosed Chlamydia cases reported to ECDC by 26 EU/EEA member states was 184 per 100 000 population (385 307 cases) [1]. Untreated, Chlamydia can cause serious sequelae among women, including pelvic inflammatory disease which can result in infertility, ectopic pregnancy and chronic pelvic pain. In men, untreated Chlamydia infection can lead to acute genital inflammation (epididymitis, epididymo-orchitis) and occasionally to sexually-acquired reactive arthritis (SARA) [2]. Chlamydia infection can facilitate the transmission of HIV [3] and is often asymptomatic for between 70 and 90 % of women, and over 50 % of men [4]. Asymptomatic carriers may remain undetected and represent a major reservoir for Chlamydia spread.

There are currently few data on the prevalence of Chlamydia in Germany as reporting of this STI is not mandatory. In a German population-based survey conducted among adolescents (2003–2006), the prevalence of Chlamydia infection was 2.2 % (95 % CI: 1.4–3.5) in girls aged 15–17 years and 0.2 % (95 % CI: 0.1–0.7) in boys aged 16–17 years [5].

In Germany, Chlamydia screening is recommended and free of charge for women under 25 years old (annually), pregnant women and women planning an abortion; there is no recommendation to screen asymptomatic men. Results from the German national surveillance network of laboratories showed that the proportion of positive tests was 3.9 % (139,632/3,540,860) among women (2008–2014). The proportion of positive tests was 11.9 % (27,720/233,692) among men [6], however physicians tend to offer Chlamydia testing mostly to symptomatic men since tests for men are only reimbursed by the statutory health system when they are symptomatic. Therefore, the proportion of positive Chlamydia tests among asymptomatic men and the prevalence among men regardless of symptom onset cannot be drawn from the German surveillance data.

Local public health authorities (LPHA) offer free anonymous HIV testing and counselling. Patients asking for an HIV test at a LPHA may have contracted other STIs such as Chlamydia. Compared to the general population, Chlamydia prevalence may be higher among persons seeking HIV testing at LPHA, since having unprotected sex is the second most frequent reason to ask for an HIV test, the first reason being having a new partner [7]. Chlamydia tests are not routinely offered to persons attending LPHA for an HIV test: in a survey conducted in 2012 among 250 LPHA offering STI/HIV counselling and testing, just 27 % offered Chlamydia testing [8]. Thus, Chlamydia tests are not routinely offered to persons attending LPHA for an HIV test. In 2012, the STI-HIT cross-sectional study commenced, with participating LPHA in North Rhine-Westphalia offering screening for Chlamydia and Gonorrhoea to patients seeking HIV testing. The focus of this article is on Chlamydia. We assessed the prevalence and factors associated with Chlamydia infection in patients seeking HIV testing at local public health authorities in order to evaluate whether Chlamydia testing should be routinely offered to them.

## Methods

### Setting and study population

In Germany, responsibility for public health is decentralised, with most public health activities being financed by rural or urban district authorities. LPHA are in charge of providing public health services at the county level. Services they offer range from vaccination, pediatric care, mental health, social medicine, hygiene and environmental health, to health promotion. According to the Infection Protection Act, LPHA should provide anonymous STI/HIV counseling and testing. The range of STI tests offered varies highly across LPHA [8].

The initial sampling design was a simple cluster sample. Each participating LPHA represented a cluster. Within a cluster, the variance in Chlamydia prevalence between participants was expected to be less than the variance in Chlamydia prevalence between clusters. This was taken into account in the calculation of the sample size with a so-called “design effect” of 2. Assuming a prevalence of 1 % (gonorrhea) up to 12 % (Chlamydia) and a power of 80 %, about 979 participants were expected to be included in the study (precision ranging from 1 to 3 %). This number was multiplied by 2 to take into account the design effect due to cluster sampling. Assuming a response proportion of 70 %, up to 2545 participants were initially expected to be included in the study.

There are 53 LPHA offering HIV testing in North-Rhine-Westphalia, the largest federal state located in the western part of Germany (~17.5 million inhabitants). All LPHA were offered to take part in the study. Between November 2012 and September 2013, 18 LPHA agreed to participate in the study and offered Chlamydia testing anonymously and free of charge to patients seeking HIV testing. All eligible patients attending LPHA were invited to participate. LPHA staff recruited study participants and offered a Chlamydia test to all of them. Chlamydia testing was independent of whether the patient actually took an HIV test. All patients 18 years old or more were eligible to participate in the study. Persons who met the exclusion criteria for participation in the study were pregnant, breast-feeding or menstruating women and persons who are already enrolled in the study. Because pregnant women are a special population requiring specific precautions, the ethical committee has implemented strict procedures for their inclusion in clinical studies and we decided not to include them in this study. To be noted that women in Germany are systematically offered free Chlamydia testing during their pregnancy. As transgenders were few and because the vast majority of sex workers were recruited by one single LPHA, we decided to exclude them from this analysis in order to focus on sub-populations represented across all LPHA.

### Data collected

#### Study questionnaire

After giving oral informed consent, study participants were administered by LPHA staff a short paper-based questionnaire providing information on their socio-demographic characteristics, sexual and HIV testing behaviours, previous STI history and clinical symptoms.

#### Diagnostic tests

Specimens were obtained through self-collected vaginal swabs and urine (men). All samples were collected at LPHA. These specimens were analysed by Transcription-Mediated Amplification (APTIMA Combo2®), a nucleic acid amplification technique targeting ribosomal RNA.

### Statistical analysis

Data analyses were performed using Stata, V.13. For categorical variables, we calculated frequencies and proportions. Chi squared tests were used when proportions were compared to detect possible differences between groups. For continuous variables, we calculated the median and presented the 1^st^ and 3^rd^ quartiles [Q1-Q3]. Due to different risk profiles in the study population, we stratified this analysis by three sub-populations: women, heterosexual men and MSM. We assessed overall and age-stratified Chlamydia prevalence and 95 % confidence intervals (CI). Using univariate and multivariable binomial regression, we estimated adjusted prevalence ratios (aPR) and 95 % CI to identify factors associated with Chlamydia infection. All variables with *P* < 0.25 in the univariate analysis were included in the multivariable model; we excluded the variables when less than 10 participants presented the outcome of interest. Overall significance was set at *P* < 0.05 in the multivariable model. We built a separate model for each group. For heterosexual men, the variable ‘number of partners’ was forced into the multivariable model as we expected it to be a key factor influencing the outcome. Similarly, for MSM, we forced ‘age group’ and ‘number of partners’ in the multivariable model. Since our study included 18 LPHA from cities in North-Rhine-Westphalia, we tried to use a mixed model to adjust for heterogeneity between the LPHA. However, this model did not significantly improve the model fit.

### Data protection and privacy

Ethical approval was obtained from Charite - University Medicine Berlin (approval number EA1/142/12). Data collected through the questionnaire as well as the laboratory results were anonymised. A unique barcode was assigned to each patient and used to link laboratory results with the corresponding questionnaire. This bar code allowed health care counsellors to get back to the patient with the test result, and to plan further appointments for counselling and treatment when necessary. After the questionnaire and laboratory data had been linked, the barcode was removed from the dataset so that the Robert Koch Institute received an anonymised dataset.

## Results

### Sociodemographic, behavioral and clinical characteristics of study participants

**Fig. 1:**
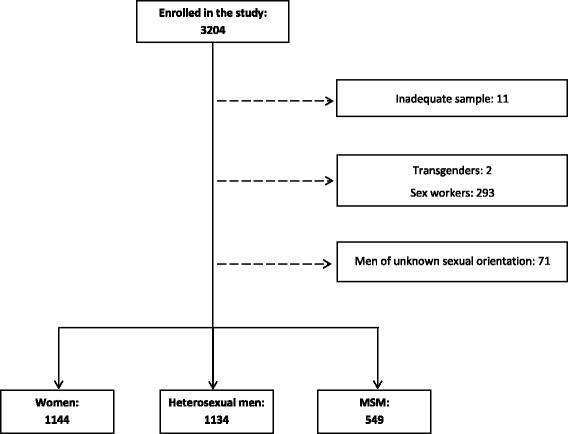
Flow chart of study participants

A total of 3204 study participants enrolled in the STI-HIT study and 2827 were included in this analysis (Fig. 1).

The population under study consisted of 40.5 % of women, 40.1 % of heterosexual men and 19.4 % of MSM. The median age was 30 years [Q1-Q3: 25–38]. Seventy-three percent completed a high-school diploma (“Abitur/Fachabitur”, 12 to 13 years of schooling) and 18 % completed a secondary-school diploma (“Realschule”, 10 years of schooling). The main reasons for getting an HIV test were having sexual contact with a person of unknown HIV status (64.1 %) and having a new partner (34.3 %). The proportion of participants having an HIV test for the first time was twice higher in women (61.5 %) and heterosexual men (60.8 %) compared to MSM (29.9 %). The median number of partners over the past 6 months was highest among MSM (3 [Q1-Q3:2–6]), followed by heterosexual men (2 [Q1-Q3:1–3]) and women (1 [Q1-Q3:1–2]). Overall, 15.4 % of study participants had symptoms at the time of counselling. Additional characteristics are summarised in Table 1.

**Table 1:** Sociodemographic, behavioral and clinical characteristics of study participants (*N* = 2827)

	Women (*N* = 1144)		Heterosexual men (*N* = 1134)		MSM (*N* = 549)	
Median age [Q1-Q3]:	29	[25–35]	31	[26–39]	32	[26–41]
	n	(%)	n	(%)	n	(%)
Level of education:						
Did not finish school	13	(1.3)	12	(1.1)	3	(0.6)
Still in school	6	(0.6)	6	(0.6)	2	(0.4)
Secondary school education (Hauptschuleabschluss)	66	(6.4)	91	(8.8)	44	(8.6)
Secondary school diploma (Realschuleabschluss)	171	(16.5)	200	(19.2)	85	(16.5)
High-school diploma (Abitur/Fachabitur)	782	(75.3)	732	(70.3)	380	(74.0)
Missing values	106		93		35	
Born in Germany:	938	(83.5)	878	(79.4)	449	(83.3)
Missing values:	20		28		10	
Having an HIV test for the first time (yes):	703	(61.5)	689	(60.8)	164	(29.9)
Missing values	15		12		1	
Reason for getting tested (multiple answers possible):						
Intercourse with a person of unknown HIV status	699	(63.3)	668	(60.4)	394	(73.0)
New partner	408	(37.1)	418	(37.8)	115	(21.3)
Intercourse with known HIV + person	13	(1.2)	10	(0.9)	51	(9.4)
Intercourse with sex workers	0		116	(10.5)	9	(1.7)
Injectable drug user	2	(0.2)	3	(0.3)	0	(0.0)
Occupational exposure	25	(2.3)	9	(0.8)	5	(0.9)
Other reason	85	(7.7)	66	(6.0)	55	(10.2)
Missing values	43		29		9	
Number of partners over the past 6 months:						
0	53	(5.9)	20	(1.8)	4	(0.9)
1	428	(47.6)	297	(26.2)	101	(23.4)
2	238	(26.4)	255	(22.5)	69	(16.0)
≥ 3	183	(20.3)	321	(28.3)	257	(59.6)
Missing values	242		241		118	
Currently has a stable partner:	620	(56.6)	692	(63.3)	257	(48.2)
Missing values	48		41		16	
Previous STI history:						
Yes	156	(14.2)	138	(12.9)	145	(27.6)
Do not know	19	(1.7)	40	(3.6)	19	(3.5)
Missing values	25		12		5	
Symptoms at time of counselling (yes):	228	(20.4)	137	(12.3)	62	(11.5)
Missing values	28		16		10	

### Prevalence of Chlamydia infection

**Fig. 2:**
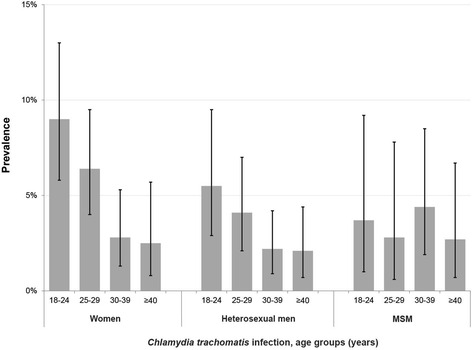
Prevalence of *Chlamydia trachomatis* infection by age group in women, heterosexual men and MSM. For each bar, the back line represents the 95 % confidence interval

The overall prevalence of Chlamydia infection was 5.3 % (61/1144; 95 % CI: 4.1 – 6.8 %) among women, 3.2 % (36/1134; 95 % CI: 2.2 – 4.4) in heterosexual men and 3.5 % (19/549; 95 % CI: 2.1–5.4) in MSM. In women, the prevalence was highest in the younger age groups, reaching 9.0 % (95 % CI: 5.8–13) among the 18–24 year-olds - the prevalence in this group differed significantly from the prevalence in the two older age groups (Fig. 2). The same pattern can be observed among heterosexual men - the highest prevalence was also found among the 18–24 year-olds (5.7 %; 95 % CI: 3.0–9.8 %) - but the higher prevalence in the younger age groups was not statistically significant when compared to the two other age groups. As to MSM, the overall pattern was different with the highest prevalence found among the 30–39 year-olds (4.4 %; 95 % CI: 1.9–8.5 %), followed by the 18–24 year-olds; the confidence intervals widely overlapped across all age groups (Fig. 2).

### Prevalence of asymptomatic infections

Among Chlamydia positive women, 76.7 % were asymptomatic. Among Chlamydia positive men, 75.0 % of the heterosexual men and 84.2 % of the MSM were asymptomatic. There was no significant difference between all three groups (*P* = 0.726).

### Factors associated with Chlamydia infection

#### Univariate binomial regression

Among women, age group, number of partners and vaginal discharge were associated with Chlamydia infection in the univariate analysis. However, only nine Chlamydia positive women reported discharge symptoms. Among heterosexual men, only age group was associated with Chlamydia infection in the univariate analysis. Among MSM, only penile discharge and painful urination were associated with Chlamydia infection in the univariate analysis. However, the absolute numbers were small among MSM who reported penile discharge (*n* = 1) or painful urination (*n* = 2). Therefore, these two variables were not included in the multivariable model.

#### Multivariable binomial regression

Among women, factors associated with Chlamydia infection in the multivariable model were young age (18–24 years versus ≥ 40 years of age, aPR: 3.0, 95 % CI: 1.2–7.8), having had more than two partners over the past 6 months (ref.: one partner, aPR: 2.1, 95 % CI 1.1–4.0) and being born abroad (aPR: 1.9, 95 % CI: 1.0–3.5). None of the other factors was associated with testing positive for Chlamydia in the final model (Table 2).

**Table 2:** Associations between variables and *Chlamydia trachomatis infection in women, heterosexual men and MSM*

Variables	Crude prevalence of Chlamydia infection %	(n/N)	Unadjusted PR	(95 % CI)	*P* value	Adjusted PR	(95 % CI)
Women (*N* = 1144)	Number of participants in the final model: *N* = 839						
Age group (years)							
≥ 40 (ref.)	2.5	(5/202)	Ref.			Ref.	
18–24	9.0	(25/279)	3.6	(1.4–9.3)	0.007	3.0	(1.2–7.8)
25–29	6.8	(22/345)	2.6	(1.0–6.7)	0.052	1.9	(0.7–4.8)
30–39	2.8	(9/318)	1.1	(0.4–3.4)	0.808	1.0	(0.3–2.9)
No. of partners							
1 (ref.)	4.4	(19/428)	Ref.			Ref.	
0	0	(0/53)	-			-	
2	6.7	(16/238)	1.5	(0.8–2.9)	0.208	1.4	(0.8–2.7)
≥ 3	9.3	(17/183)	2.1	(1.1–3.9)	0.022	2.1	(1.1–4.0)
Country of birth (ref. = Germany)	5.0	(47/938)	Ref.				
Abroad	7.5	(14/186)	1.5	(0.8–2.7)	0.166	1.9	(1.0–3.5)
Vaginal discharge (ref. = no)	4.9	(52/1073)	Ref.				
Yes	12.7	(9/71)	2.6	(1.3–5.1)	0.005	-	-
Reason for getting tested							
(ref. = not a reason for getting tested)	4.2	(17/404)	Ref.				
Intercourse with a person of unknown HIV status	6.2	(43/697)	1.5	(0.9–.2.6)	0.126	-	-
(ref. = not a reason for getting tested)	5.3	(57/1076)	Ref.				
Occupational exposure	12	(3/25)	2.3	(0.8–6.9)	0.131	-	-
Heterosexual men (*N* = 1134)	Number of participants in the final model: *N* = 854						
Age group (years)							
≥ 40 (ref.)	1.8	(5/274)	Ref.			Ref.	
18–24	5.7	(12/210)	3.1	(1.1–8.8)	0.029	4.1	(1.3–13)
25–29	4.2	(12/288)	2.3	(0.8–6.4)	0.116	2.0	(0.6–6.7)
30–39	1.9	(7/362)	1.1	(0.3–3.3)	0.920	1.2	(0.3–4.2)
No. of partners							
1 (ref.)	3.4	(10/297)	Ref.			Ref.	
0	0.0	(0/20)	-			-	
2	3.5	(9/255)	1.0	(0.4–2.5)	0.917	1.1	(0.4–2.7)
≥ 3	3.4	(11/321)	1.0	(0.4–2.4)	0.967	0.9	(0.4–2.2)
Reason for getting tested (ref. = not a reason for getting tested)	2.3	(10/437)	Ref.			Ref.	
Intercourse with a person of unknown HIV status	3.6	(24/668)	1.6	(0.8–3.3)	0.224	1.7	(0.7–3.9)
MSM (*N* = 549)	Number of participants in the final model: *N* = 358						
Age group (years)							
≥ 40 (ref.)	2.7	(4/150)	Ref.			Ref.	
18–24	3.7	(4/108)	1.4	(0.4–5.4)	0.637	1.7	(0.4–6.6)
25–29	2.8	(3/109)	1.0	(0.2–4.5)	0.967	0.4	(0.04–3.4)
30–39	4.4	(8/182)	1.6	(0.5–5.3)	0.412	1.3	(0.4–4.3)
No. of partners							
1 (ref.)	2.0	(2/101)	Ref.			Ref.	
0	0.0	(0/4)	-			-	
2	0.0	(0/69)	-			-	
≥ 3	5.1	(15/431)	2.6	(0.6–11)	0.211	2.8	(0.6–12)
Penile discharge (ref. = no)	3.3	(18/546)	Ref.				
Yes	33.3	(1/3)	10.1	(1.9–53)	0.006	-	-
Painful urination (ref. = no)	3.2	(17/534)	Ref.				
Yes	13.3	(2/15)	4.2	(1.1–17)	0.041	-	-

Among heterosexual men, young age was the only factor associated with Chlamydia infection in the multivariable model (18–24 years versus ≥ 40 years of age, aPR: 4.1, 95 % CI: 1.3–13) (Table 2). Among MSM, age was not found to be associated with Chlamydia infection (18–24 years versus ≥ 40 years of age, aPR: 1.7, 95 % CI: 0.4–6.6), nor was number of partners (ref.: one partner, aPR: 2.8, 95 % CI 0.6–12) (Table 2).

## Discussion

In our study, the prevalence of Chlamydia infection was high among women under 30 years-old and heterosexual men under 25 years (point estimate above 5 %). Chlamydia prevalence was also above 4 % among the 25–29 year-olds in heterosexual men, and among the 30–39 year-olds in MSM. Looking at point estimates, the overall pattern was similar among women and heterosexual men - a decrease in the prevalence was observed as age increased - and differed with that of MSM for whom age did not seem to be a key factor associated with urethral Chlamydia infection. This was confirmed in the multivariable analysis: the prevalence of Chlamydia infection was significantly higher among women and heterosexual men aged 18 to 24 years whereas age group was not associated with Chlamydia infection among MSM in the univariate and multivariable analysis. Across all three groups, the vast majority of participants who tested positive for Chlamydia were asymptomatic. As previously highlighted, only testing of symptomatic men is reimbursed by the German statutory health system. Since Chlamydia infection is often asymptomatic in men as well, the vast majority of positive tests is missed when testing symptomatic men only, fueling the reservoir of asymptomatic carriers. In this study, above three quarters of Chlamydia infection would have been missed among men if only symptomatic men had been tested. This raises the question as to Chlamydia screening not only among asymptomatic women but also among asymptomatic men.

The presently scarce data on Chlamydia prevalence in Germany include two cross-sectional studies: one was conducted among adolescents in the general population [5], the other one was conducted among MSM attending local health offices, STI clinics and private medical practices in Germany [9]. In the German context, we were only able to compare our results for specific age groups with the proportion of positive tests based on data from the surveillance network of laboratories in North Rhine-Westphalia. However, these surveillance data cannot be interpreted as a measure of Chlamydia prevalence as the proportion of positive tests is highly dependent on screening recommendations.

Our study provides data as to Chlamydia prevalence among persons attending LPHA for an HIV test, which was previously unknown. Prevalence of Chlamydia infection was assessed in three sub-populations (women, heterosexual men and MSM) in order to distinguish specific recommendations for each group.

Chlamydia screening in Germany is recommended and reimbursed by the statutory health system for women under 25 years old. Our results showed that Chlamydia prevalence reached 9.0 % among 18–24 year-old women seeking HIV testing at a LPHA. In comparison, the proportion of positive Chlamydia tests in that same age group was 4.8 % based on data from the national surveillance network of laboratories for North Rhine-Westphalia [6]. Similarly, the overall Chlamydia prevalence estimated among women (5.3 %) was also higher compared to data from the national surveillance network of laboratories for North Rhine-Westphalia (proportion of positive tests: 3.8 % [3.7–3.8 %], 2008–2014).

For men, the prevalence estimated in this study can hardly be compared with the proportion of positive tests from the surveillance data as tests for men are only reimbursable when they are symptomatic. In the case of men, the German surveillance data are likely to overestimate Chlamydia prevalence since only symptomatic men tend to be tested.

In this study, men were tested for Chlamydia using urine samples. Therefore, we could only estimate the prevalence of urethral Chlamydia infection. In a cross-sectional study conducted among MSM in Germany [9], prevalence of urethral Chlamydia was 3.4 % (95 % CI: 2.0–4.7 %) which is consistent with our results. In that same study, Chlamydia prevalence was 1.5 % (95 % CI: 1.0–2.0 %) in pharyngeal swabs and 8.0 % (95 % CI: 6.8–9.2 %) in rectal specimen. Seventy-five percent of Chlamydia infections were exclusively rectal, supporting other evidence that only a minority of prevalent infection would be detected when testing MSM for urethral infections alone [9–11]. Besides, rectal and pharyngeal infections are often asymptomatic [12]. The study conducted in Germany highlighted the need for pharyngeal and rectal Chlamydia testing for MSM, in addition to screening for urethral infection [9].

There is currently no recommendation to screen asymptomatic men in Germany. In France, systematic Chlamydia screening of all men under 30 attending free and anonymous screening centers, information, diagnosis and screening centers for sexually transmitted infections, or family planning and education centers, was recommended by the National Agency for Health Accreditation and Evaluation (ANAES) in 2003 [13]. In the United Kingdom, in primary care, opportunistic screening is recommended for those in whom the prevalence is known to be the highest: the under 25 years old (women and men), those with more than two partners over the past 12 months or with a recent change of sexual partner [4].

Cost-effectiveness studies can help inform policy makers with respect to possible Chlamydia screening strategies. However, there are challenges in assessing the cost-effectiveness of Chlamydia screening. In a review of published cost-effectiveness studies focusing on asymptomatic women under 30 years old in a primary care setting, a threshold of 3 % was identified as the prevalence from which routine screening for Chlamydia is cost-effective [14]. However, this threshold is questioned as the probability of developing pelvic inflammatory disease is yet to be precisely established. The natural history of untreated Chlamydia cannot be directly observed in humans for ethical reasons. In 2012, a mathematical modeling study simulating results of published randomised trials estimated that 10 % (95 % CI: 7–13 %) of chlamydia infections progress to pelvic inflammatory disease over one year, assuming constant progression of the infection to the disease or progression at the end [15]. Further research is needed in order to assess the cost-effectiveness of screening asymptomatic men for Chlamydia and to decide which subgroups among men may be included as part of a wider Chlamydia screening strategy.

The results reported here have a number of limitations. We do not have information on the response proportion, therefore we do not know whether those who did not take part in the study differed from those who did with respect to Chlamydia infection: selection bias cannot be ruled out. Most of the local public health authorities who took part in this study were located in large cities of North Rhine-Westphalia and are more likely to be representative of LPHA in large cities. Few rural LPHA participated as they had few clients. The results reported apply to the federal state of North Rhine-Westphalia and cannot be extrapolated to the whole of Germany. In this study, information on condom use was not collected. It is possibly a key factor associated with Chlamydia infection as consistent condom use has shown to be effective in reducing the risk of STI such as Chlamydia [16]. Information on alcohol and drug consumption were not collected and may also be factors influencing the outcome [5, 17]. For variables with more than 10 % of missing values, multiple imputation of the missing data is an option if the data are missing at random. In our study, the proportion of missing values for ‘number of partners’ was above 10 % but we could not exclude that it was missing not at random. Lastly, self-collected vaginal swabs have been identified as the specimens of choice when screening women for Chlamydia [18, 19]. Compared to vaginal swabs in women, use of urine specimen in men may have led to less sensitive detection of Chlamydia infection among men.

In Germany, the coverage of the national Chlamydia screening programme is low, approximately 12 % of the eligible population is reached [20]. Routine Chlamydia testing of women and possibly men who seek HIV testing at a LPHA, prioritising groups with a higher prevalence, provides an opportunity to detect and treat asymptomatic infections in individuals who might not seek Chlamydia testing otherwise. Besides, patients seeking an HIV test following unprotected sex are at risk of multiple STIs . The potential of voluntary counselling and testing could be maximised by providing tests for Chlamydia, and possibly other STIs, integrated in a single service visit [21, 22].

## Conclusions

LPHA offering HIV tests should consider offering routine Chlamydia testing to women under 30 years. In addition, having multiple partners and being born abroad were identified as independent factors associated with Chlamydia infection, these two groups may also be considered for routine Chlamydia testing. Being born abroad is likely to be a proxy reflecting higher risk behaviours among patients born abroad seeking HIV testing at LPHA. It may also reflect less access to testing and treatment in the country of birth.

If testing of asymptomatic men visiting LPHA for an HIV test is considered in Germany, our results suggest offering routine Chlamydia testing to heterosexual men under 25 years old or even under 30, depending on where the screening threshold is set.

For MSM, young age did not seem to be a key factor associated with urethral Chlamydia infection. We cannot draw specific recommendations based on our study as we estimated the prevalence of urethral Chlamydia infection only, leaving out rectal and pharyngeal infections. Future studies would have to include pharyngeal and rectal swabs in order to evaluate whether routine Chlamydia testing is warranted for MSM in this setting.

## Abbreviations

CI: Confidence interval
HIV: Human Immuno-deficiency Virus
LPHA: Local Public Health Authorities
MSM: Men who have Sex with Men
Q1-Q3: 1st quartile – 3rd quartile
STI: Sexually Transmitted Infection
